# What happens when you tell someone you self-injure? The effects of disclosing NSSI to adults and peers

**DOI:** 10.1186/s12889-015-2383-0

**Published:** 2015-10-09

**Authors:** Penelope Hasking, Clare S. Rees, Graham Martin, Jessie Quigley

**Affiliations:** School of Psychology and Speech Pathology, Curtin University, GPO Box U1987, Perth, WA 6845 Australia; Department of Psychiatry, Monash University, Melbourne, Australia; Centre for Clinical Psychiatry and Neuroscience, The University of Queensland, Queensland, Australia

**Keywords:** NSSI, Help-seeking, Adolescents

## Abstract

**Background:**

Non-suicidal self-injury (NSSI) is associated with significant adverse consequences, including increased risk of suicide, and is a growing public health concern. Consequently, facilitating help-seeking in youth who self-injure is an important goal. Although young people who disclose their NSSI typically confide in peers and family, it is unclear how this disclosure and related variables (e.g. support from family and friends, coping behaviours, reasons for living) affect help-seeking over time. The aim of this study was to advance understanding of the impact of disclosure of NSSI by young people and to investigate these effects over time.

**Methods:**

A sample of 2637 adolescents completed self-report questionnaires at three time points, one year apart.

**Results:**

Of the sample, 526 reported a history of NSSI and 308 of those who self-injured had disclosed their behaviour to someone else, most commonly friends and parents.

**Conclusions:**

Overall, we observed that disclosure of NSSI to parents facilitates informal help-seeking, improves coping and reduces suicidality, but that disclosure to peers might reduce perceived social support and encourage NSSI in others. We discuss these findings in light of their clinical and research implications.

## Background

Non-suicidal self-injury (NSSI) is a challenging, yet prevalent, behaviour which typically begins during adolescence [1] and is related to difficulties with emotion regulation [2]. Although distinct from suicidal behaviour, a prolonged history of NSSI is a salient risk factor for later suicidal thoughts and behaviour [3]. For the 18 % of young people who engage in NSSI, the importance of seeking help to address psycho-social concerns is clear [1, 4]. Early help-seeking for emotional difficulties is central to developing resilience among adolescents, reducing subsequent NSSI and suicide. Unfortunately, young people who self-injure avoid help, a phenomenon known as help negation [5]. Help negation manifests in the behavioural refusal or avoidance of obtaining and engaging in available help services, as well as the cognitive relationship between reported symptoms and intention to seek help, from either formal or informal sources of support [6].

Help negation is influenced by a number of factors that work to facilitate or deter people from engaging in help seeking. Possibly one of the most important barriers to help seeking is one’s comfort with self-disclosure, with evidence implicating low comfort with self-disclosure in help negation [7]. Despite increasing visibility, NSSI is still highly stigmatised and met with fear and confusion by parents e.g. [8], school staff [9, 10] and medical professionals [11]. Adolescents report that NSSI is often misinterpreted as a suicide attempt [12], further deterring disclosure of the behaviour. While there is an assumed benefit in disclosing NSSI to adults, fearful or negative reactions from parents and family members may exacerbate psychological distress among youth, increase secrecy and limit help-seeking. Such reactions would also have deleterious effects on the level of perceived support available to the young person, further increasing a sense of isolation. Finally, the way family and friends react significantly impacts the family and social relationships [8, 13].

Not surprisingly, young people prefer to disclose NSSI to friends rather than adults [14]. Seeking help from informal sources, including peers and family, facilitates formal help-seeking [15] and could result in improved psychosocial functioning and coping over time. Yet, there is concern that discussing NSSI within peer groups may increase contagion [14], with evidence that knowing a peer who self-injures increases the likelihood that a young person will self-injure [16]. As such, disclosing to parents and other adults might offer a more positive avenue for future help-seeking.

In summary, while there are likely benefits in disclosing NSSI to parents and other adults, how they react is paramount to future help-seeking [17]. Similarly, while disclosure to peers might facilitate help-seeking it may also exacerbate or encourage NSSI within the peer group. Facilitating help-seeking for NSSI, and assisting parents and families to effectively respond to disclosures, requires a better understanding of the effects of disclosure on the young person. Previous work has confirmed preferential help-seeking from friends, however no studies have explicitly explored disclosure of NSSI or the impact this may have on the young person. In this study we: 1) examined differences between youth who disclosed NSSI and those who did not with regard to help-seeking behaviour, coping strategies, social support, reasons for living (a proxy of suicidality) and severity of NSSI; 2) explored the effect of disclosure on changes in help-seeking behaviour, coping strategies, social support, reasons for living and severity of NSSI over a two year period; and 3) explored whether these effects were different according to whether the confidante was a peer or an adult. Although this study is largely exploratory, we expected that youth who disclosed their NSSI would report better psychosocial functioning and that disclosure to adults would be more beneficial than disclosure to peers. Finally, given that longer histories of NSSI [3], and help-negation [6], are each related to suicidal behaviour we propose that failure to disclose NSSI might relate to later suicidality.

## Method

### Participants

At baseline, 2637 Australian high school students (aged 12–18 years) completed questionnaires as part of a larger project e.g. [18]. An additional 354 students participated in the study for the first time at Time 2 (one year later), and 152 students joined for the first time at Time 3 (2 year follow-up). Of these students, 45.3 % (*n* = 1424) completed questionnaires at all time points, and 30.5 % (*n* = 959) completed questionnaires in at least two waves of data collection, generally consistent with longitudinal studies examining suicidality [19]. Primary reasons for attrition included students not attending scheduled questionnaire administration, school transfers and student/parent withdrawal of consent. Mean age of participants at baseline was 13.93 years (*SD* = .99).

Most participants were born in Australia (89.3 %); 2.3 % identified as Aboriginal or Torres Strait Islander, representative of the indigenous population in Australia [20]. Participants from areas of higher socio-economic advantage were oversampled relative to the general Australian population [21]. Of the sample, 6.4 % reported that a doctor had told them they had an emotional or behavioural problem, most commonly mood and/or anxiety disorders; 25 % reported seeing a mental health professional, most commonly a counsellor.

### Materials

#### Self-Harm Behaviour Questionnaire – Part A (SHBQ) [22]

On the SHBQ respondents indicate if they have ever engaged in NSSI, and if so, describe how they injured themselves, age of onset, recency, frequency and medical severity (from ‘not at all serious’ to ‘life threatening’). NSSI was defined for respondents as “hurt yourself on purpose without trying to kill yourself”. Respondents who indicated they engaged in NSSI with intent to die, or where method of NSSI was ambiguous (e.g. overdose), were not classified as engaging in NSSI (*n* = 22). Participants were also asked whether they had ever seriously thought about taking their life, and if they had ever tried to take their life. The SHBQ has excellent internal consistency (α = .95), including in adolescent samples [23]. Alphas for the present study were high (α = .88–.93).

#### Actual help-seeking questionnaire [24]

Participants were asked whether they had sought help in the past two weeks from each of 10 sources (friend, boy/girlfriend, parent, mental health worker etc.) for an emotional and/or behavioural problem, and asked to name the problem for which they sought help. The AHSQ is an accurate measure of past help-seeking behaviour, especially when used together with the General Help-Seeking Questionnaire [24].

#### General help-seeking questionnaire [25]

The General Help-Seeking Questionnaire (GHSQ) assesses future intentions to seek help for a hypothetical emotional or behavioural problem. Participants indicated their intention to seek help from each of ten sources on a 7-point Likert scale ranging from 1 (“extremely unlikely”) to 7 (“extremely likely”). The scale exhibits sound internal consistency (Cronbach’s α = .70) and test-retest reliability (r = .86), modest predictive validity and strong convergent evidence for criterion validity [25]. The GHSQ had a Cronbach’s alpha of .66 in the current sample.

#### Adolescent Coping Scale (ACS) [26]

The short form of the ACS consists of 18 items assessing three primary factors: problem solving, reference to others and non-productive coping. The scale shows acceptable test-retest reliability, and predictive validity [26]. Cronbach’s alphas in our sample were: non-productive = .74; problem-solving = .76; reference to others = .38.

#### Multidimensional Scale of Perceived Social Support (MSPSS) [27]

Three subscales of this measure assess perceived support from family, friends and significant others [27]. Each statement is scored on a 7-point Likert scale (1 = very strongly disagree, 7 = very strongly agree). The MSPSS has strong internal consistency for adolescents with alphas .81–.92 for the three subscales, good construct validity, discriminant validity and test-retest reliability [28]. With the current sample, perceived family support, friend support and significant other support demonstrated Cronbach’s alphas of .90, .91 and .93 respectively.

#### Brief Reasons for Living Questionnaire – for Adolescents (BRFL-A) [29]

The BRFL-A is a 14-item measure that assesses reasons adolescents endorse for *not* ending their lives, rated on a 6-point scale (1 = not at all important; 6 = extremely important). Five factors assess: moral objections, fear of social disapproval, survival and coping beliefs (i.e. belief in being able to find other solutions to problems), responsibility to family and fear of suicide. Confirmatory factor analysis supports the 5-factor structure, and the measure has evidenced the ability to distinguish suicidal from non-suicidal adolescents [29]. In the current study: fear of disapproval α = .62; moral objection α = .67; survival and coping beliefs α = .74; family responsibility: α = .72; fear α = .71.

### Procedure

After receiving ethical approval from Monash University, the University of Queensland and the educational jurisdictions who oversee access to schools, schools in five Australian states/territories were invited to participate. Both single-sex and co-educational schools were approached, however, more all-girl schools than all-boy schools agreed to participate (all-girl schools = 11; all-boy schools = 4; co-educational schools = 25), resulting in an over-representation of girls in the sample (Time 1 = 68.0 %, Time 2 = 70.7 %, Time 3 = 71.2 %) [30]. Information sheets and consent forms were distributed to all parents/guardians of students in the first three or four years of school. At baseline, 3117 students received parental consent to participate, a rate (21 %) consistent with previous Australian studies requiring active parental consent [31].

To protect confidentiality and enable identification in the event responses raised concerns about immediate risk, a unique code was derived for each student who participated in the study. Participants completed the questionnaire on school grounds; researchers were present to clarify questions. Participants took approximately one hour to complete the questionnaire, and on completion, received an information pack with printed materials about mental health issues and resources in the community. The same procedure was used at all time points.

### Data analysis

Data was not missing completely at random (Little’s MCAR; *χ*^2^ (4677) = 4966.58, *p* < .01), but attrition analyses suggested data was at least missing at random (MAR) [32]. Multiple imputation was used to replace less than 10 % missing data within each wave. Analyses with imputed and complete case data revealed minimal discrepancies; imputed data are reported.

Relationships with continuous dependent variables were tested with a series of doubly multivariate analyses of variance, in which disclosure of NSSI (yes/no) was entered as a between-subjects factor and changes in the continuous variables over time as within-subjects factors. Four MANOVAs assessed the effects of disclosure and changes over time on four sets of dependent variables, grouped thematically: 1. Help-seeking behaviour (past help-seeking and future help-seeking intentions), 2. Coping strategies (problem solving, reference to others, non-productive coping), 3) Social support (from family, friends, significant others), 4. Reasons for living (moral objections, fear of social disapproval, survival and coping beliefs, responsibility to family, fear of suicide). A final mixed-model ANOVA assessed changes in severity of NSSI (assessed with the total score from the SHBQ). These were repeated, selecting only participants who had disclosed their NSSI, to assess whether there were differential consequences of confiding in peers or adults. Means across time are presented in Table 1. Multivariate effects for each MANOVA are presented in Table 2.

**Table 1:** Mean scores over time on each dependent variable, according to whether youth disclosed their NSSI or not

	Disclosure of NSSI						No disclosure of NSSI					
	Baseline		Time 2		Time 3		Baseline		Time 2		Time 3	
	Mean	sd	Mean	sd	Mean	sd	Mean	sd	Mean	sd	Mean	sd
NSSI severity	9.24	2.62	8.22	4.48	8.40	4.42	9.64	2.61	6.29	4.53	7.29	4.00
Actual help-seeking	3.10	1.77	3.05	1.59	3.49	1.56	2.61	1.52	2.61	1.52	2.89	1.47
Help-seeking intentions	33.24	8.59	28.30	7.70	34.84	9.03	31.68	11.51	26.35	6.74	33.44	8.61
Productive coping	54.45	13.33	54.44	13.92	56.39	13.33	57.64	11.05	59.50	10.34	59.33	10.12
Reference to others	48.35	13.17	48.09	14.97	47.44	14.98	45.56	14.86	47.10	15.42	46.72	12.69
Non-productive	60.32	10.08	58.53	11.01	58.95	11.48	59.37	10.68	56.88	11.68	55.30	11.17
Social support - friend	21.40	5.75	21.06	5.49	20.71	5.48	20.21	5.75	20.67	5.33	20.52	5.28
Social support - family	18.61	5.96	19.24	5.88	19.28	5.77	18.57	5.77	19.37	5.48	18.67	5.68
Social support - sig other	22.64	5.15	21.94	5.38	21.14	5.72	20.52	5.89	21.10	5.63	20.46	5.93
RFL fear of disapproval	10.31	3.90	10.25	3.93	11.03	4.09	11.13	3.92	10.55	4.09	10.16	3.58
RFL moral reasons	7.08	3.99	7.32	4.16	7.44	4.23	8.72	4.38	8.45	4.61	8.51	4.53
RFL coping beliefs	11.72	3.61	12.42	3.70	12.33	3.49	12.31	2.87	13.18	2.97	12.33	2.89
RFL family responsibility	12.94	4.03	13.56	3.84	13.76	3.60	13.67	3.30	13.82	3.41	13.82	3.61
RFL fear of suicide	6.81	3.47	6.82	3.76	7.14	3.64	7.73	3.32	7.69	3.24	7.24	3.10

**Table 2:** Omnibus results for multivariate tests on combined dependent variables

	Between group effect				Within group effect				Interaction effect			
	λ	F	p	η^2^	λ	F	p	η^2^	λ	F	p	η^2^
Help-seeking^a^												
Disclosure vs no disclosure	.04	3.55	.03	.04	.27	14.34	.000	.27	.003	.10	.98	.003
Peers vs adults	.01	1.09	.34	.007	.10	8.46	.000	.10	.03	2.15	.08	.03
Coping^b^												
Disclosure vs no disclosure	.05	7.82	.000	.025	.11	10.27	.000	.11	.03	2.77	.01	.03
Peers vs adults	.04	3.93	.01	.04	.07	3.96	.001	.07	.06	3.14	.01	.06
Social support^c^												
Disclosure vs no disclosure	.02	3.68	.01	.02	.04	4.02	.001	.04	.03	2.73	.01	.03
Peers vs adults	.04	3.59	.01	.04	.14	8.15	.000	.14	.06	3.31	.004	.06
Reasons for living^d^												
Disclosure vs no disclosure	.04	1.93	.09	.04	.09	2.18	.02	.09	.06	1.56	.12	.06
Peers vs adults	.03	1.59	.16	.03	.17	5.90	.000	.17	.06	2.02	.03	.06
NSSI severity												
Disclosure vs no disclosure	n/a	6.19	.01	.02	.83	32.02	.000	.17	.94	9.26	.000	.06
Peers vs adults	n/a	.23	.63	.001	.92	8.17	.000	.08	.95	5.57	.004	.05

^a^ Actual help-seeking and General help-seeking scales

^b^ Problem solving, Reference to Others and Non-Productive Coping scales

^c^ Social support from Family, Friends and Significant Others

^d^ Comprising all subscales of the Reasons for Living Questionnaire

## Results

At baseline 526 (10.2; 28 % male; 72 % female) adolescents reported a history of NSSI. The most frequent methods were cutting, self-battery and severe scratching. Among those who self-injured the average lifetime frequency was 16.92 episodes (range 1–300 times). Average age of first episode was 12.53 years (*sd* = 2.45). The majority of injuries were rated as ‘not at all serious’ or needing first aid (88 %), with 31 participants stating that at least once they had to see a doctor as a result of NSSI.

Of the sample, 70 % had sought help from someone for an emotional or behavioural problem, most often friends (50.2 %), parents (44.3 %), or a teacher (11 %). Only 6.6 % had sought help from a mental health professional, and 2.6 % from a family doctor. Among self-injurers, 83 % had sought help, preferentially from friends (68 %), parents (30 %), and teachers (13 %). When asked what problem they were seeking help for, participants cited family problems, fights with friends and problems at school as primary sources of distress; only three explicitly cited NSSI as the problem for which they sought help.

### Disclosure of NSSI

Of the sample, 308 reported at baseline that they confided their NSSI to someone else (58.56 %). Confidantes included: friends *n* = 106 (68.83 %), parents *n* = 41 (26.62 %), mental health workers *n* = 21 (13.64 %), general practitioners *n* = 12 (7.79 %), boy/girlfriends *n* = 18 (11.69 %), siblings/cousins *n* = 5 (3.25 %), and teachers: *n* = 5 (3.25 %). No gender, *χ*^2^(1, *N* = 249) = 3.42, *p* = .18, or age, *t*(241) = 1.76, *p* = .08, differences were observed according to whether NSSI was disclosed, thus these were not controlled in remaining analyses.

Participants who self-injured were more likely to report a history of suicide ideation, *χ*^2^(1, *N* = 2535) = 372.46, *p* < .001, and attempt, *χ*^2^(1, *N* = 2509) = 169.05, *p* < .001. Similarly, NSSI at baseline was associated with subsequent ideation and attempt (all *p* < .001). Disclosure of NSSI was not related to suicide ideation, at any time point (all *p* > .05).

In order to determine if there were differential consequences associated with confiding in peers or adults, participants were classified based on the first response provided to: ‘Who did you tell?’ Friends, siblings, boy/girlfriends and online friends were classed as peers, while parents, teachers, mental health workers and GPs were classed as adults. Where status as a peer or adult was unclear (e.g. cousins) confidantes were excluded from analyses. Classified this way, 67.8 % told a peer and 32.2 % told an adult. No gender, *χ*^2^(1, *N* = 304) = .38, *p* = .54, or age, *t*(302) = .94, *p* = .35, differences were observed according to whom NSSI was disclosed, thus these were not controlled in remaining analyses.

### NSSI in the social group

At baseline, adolescents who disclosed their NSSI to someone were more likely to have friends who self-injured, *χ*^2^(1, *N* = 526) = 28.01, *p* < .001 (73.9 % vs 50.0 %), and have a greater number of friends who self-injured, *t*(470) = 5.16, *p* < .001 (mean = 2.84 vs 1.68). Among those who did not report having friends who self-injure at baseline, disclosure of their own NSSI was related to an increased likelihood of reporting having friends who self-injured at Time 2, *χ*^2^(1, *N* = 102) = 17.26, *p* < .001, and Time 3, *χ*^2^(1, *N* = 78) = 8.72, *p* = .003. Choice to disclose to a peer or adult was not related to the probability of having a friend who self-injures (all *p* > .05), number of friends self-injuring (*p* = .23), or acquiring friends who self-injure over the course of the study (all *p* > .05).

### Help-seeking behaviour

At baseline, those who had disclosed their NSSI were more likely to report previously seeking help for an emotional and/or behavioural problem from a boy/girlfriend, *χ*^2^(1, *N* = 526) = 11.55, *p* = .001, but did not differ in help-seeking from any other source. Not surprisingly, adolescents who confided their NSSI to adults were more likely to report seeking help from a parent for an emotional and/or behavioural problem, *χ*^2^(1, *N* = 200) = 7.92, *p* = .005, but no other differences in sources of support were observed.

Both disclosure and time had an effect on actual help-seeking and help-seeking intentions (Table 2). Overall, actual help-seeking did not change from baseline to Time 2 (*p* > .05), but increased by Time 3, *F*(1157) = 6.29, *p* = .01. Help seeking intentions decreased over the first year, *F*(1157) = 24.04, *p* < .001, but increased by Time 3, *F*(1157) = 29.47, *p* < .001. In those who had disclosed their NSSI, significant effects of time were evident on help-seeking in multivariate analyses, but there was no effect of whether disclosure was to peers or adults, and no interaction. Both actual help-seeking, *F* (2604) = 10.73, *p* < .001, and intentions to seek help increased over time, *F* (2604) = 4.51, *p* = .01, but while intentions demonstrated an initial increase *F* (1302) = 5.27, *p* = .02, they plateaued at Time 3, *F* (1302) = 3.78, *p* = .05. Conversely, actual help-seeking decreased initially, *F* (1302) = 9.04, *p* = .003, and then increased at Time 3, *F* (1302) = 12.28, *p* = .001 (Table 2).

At baseline, among those who had engaged in NSSI only 36 (14.46 %) had not engaged the help of anyone for an emotional and/or behaviour problem. Among these participants, there was no relationship between disclosing NSSI and the probability of seeking help for an emotional and/or behavioural problem at Time 2, *χ*^2^(1, *N* = 36) = .49, *p* = .48, or Time 3, *χ*^2^(1, *N* = 36) = .07, *p* = .79. Participants who disclosed to peers were more likely to report actual help-seeking at Time 3 than those who disclosed to adults, *χ*^2^(1, *N* = 36) = 5.39, *p* = .02, specifically from friends, *χ*^2^(1, *N* = 36) = 8.00, *p* = .001.

### Coping strategies

Main effects of disclosure, time and an interaction between disclosure and time were observed on the combined coping variables. Specifically, the group that disclosed their NSSI reported less problem focused coping*, F*(1, 484) = 11.99, *p* < .001, and more non-productive coping, *F* (1484) = 5.31, *p* = .02, than those who did not disclose. Non-productive coping, *F*(2968) = 4.68, *p* = .009, incrementally decreased at each time point (all *p* < .001), while problem-solving, *F* (2968) = 6.81, *p* = .001, was stable from baseline to Time 2, *F* (1484) = 3.92, *p* = .05, but increased at Time 3, λ = .05, *F* (1484) = 10.49, *p* = .001. An interaction was only noted for non-productive coping; decreasing over time for both groups, but the initial effect over the first year was stronger for those who disclosed their NSSI (*p* = .02 vs *p* = .001).

Among those who disclosed NSSI, main effects of the target of disclosure (i.e. peer vs adult), main effects of time and interaction effects were observed on the combined dependent variables. Specifically, those who disclosed to peers reported more non-productive coping than those who disclosed to adults, *F* (1302) = 4.93, *p* = .03. Problem focused coping did not change from baseline to Time 2 (*p* > .05) but increased at the third data collection point, *F* (1302) = 13.49, *p* < .001. Similarly, non-productive coping was initially stable, (*p* > .05) but decreased by Time 3, *F* (1302) = 9.56, *p* = .002. Interaction effects were only evident for problem focused coping; adolescents who confided in peers demonstrated no growth in problem focused coping initially (*p* > .05) but improved by the final data collection point, *F* (1,97) = 5.63, *p* = .02, while those who confided in adults demonstrated steady growth in problem focused coping across Time 2, *F* (1,97) = 4.02, *p* = .05, and Time 3, *F* (1,97) = 8.85, *p* = .004.

### Social support

Changes over time and an interaction between disclosure and time were evident on the combined social support variables (Table 2). Social support from family increased from baseline to Time 2, *F*(1242) = 4.34, *p* = .04, but then decreased at Time 3, *F*(1242) = 4.78, *p* = .03. Interaction effects were observed on support from family, *F*(4484) = 2.45, *p* = .045, friends, *F*(4484) = 2.57, *p* = .04, and significant others, *F*(4484) = 3.49, *p* = .008. Support from family initially increased, then plateaued for both groups (disclosure group *p* = .045; no disclosure *p* = .005). Although not significant, support from friends decreased when NSSI was disclosed but increased when the behaviour was concealed. Similarly, support from significant others tended to decline over time for those who confided their NSSI (*p* = .025), while support increased (*p* = .025) for those who did not reveal their behaviour.

When considering differential effects of disclosure to peers and adults, disclosure, time and an interaction were evident on the combined dependent variables (Table 2). Adolescents who disclosed NSSI to peers reported more support from friends than those who confided in adults, *F*(1302) = 6.06, *p* = .01. Across the groups, family support increased from baseline to Time 2, *F*(1302) = 22.29, *p* < .001, and again at Time 3, *F*(1302) = 9.42, *p* = .002. Interaction effects were only evident when considering social support from friends; among those who confided in friends, perceived support from friends decreased from baseline to Time 2, *F* (1,97) = 10.71, *p* = .001, and again at Time 3, *F* (1,97) = 4.72, *p* = .03. Conversely, among those who confided in adults, perceived support from friends increased from baseline to Time 2, *F* (1,97) = 5.23, *p* = .02, and then stabilised (*p* > .05).

### Reasons for living

No main effect of disclosure or an interaction was observed on the combined reasons for living. There was an effect of time, but univariate analyses revealed the ‘survival and coping beliefs’ subscale to be the only significant variable, *F* (2482) = 9.40, *p* < .001. Significant increases in coping beliefs were evident at Time 2, *F*(1241) = 12.72, *p* < .001, and again at Time 3*, F*(1241) = 5.61 *p* = .01.

Despite not exerting an effect on the combined dependent variables, an interaction affecting the ‘fear of social disapproval’ subscale was evident in univariate analyses, *F*(2, 482) = 4.50 *p* = .01. While fear of social disapproval increased among participants who disclosed their NSSI, *F*(2, 176) = 3.23, *p* = .03, particularly from Time 2 to Time 3 (*p* = .01), there was no change among participants who did not disclose their behaviour, *F*(2, 176) = 2.12, *p* = .12.

Among those who disclosed their behaviour, reasons for living changed over time, and an interaction with disclosure to a peer or adult was observed. Across the sample scores on all subscales, except fear of suicide, increased over time (all *p* < .01). While moral objections, responsibility to family and survival and coping beliefs all demonstrated incremental gains at each time point (all *p* < .05), social disapproval did not change from baseline to Time 2 (*p* < .05) but increased by Time 3, *F* (1302) = 8.00, *p* = .005. An interaction effect was only observed for coping beliefs, *F* (2604) = 5.64, *p* = .004; no change was observed among those who confided in peers (all *p* > .05), while those who confided in adults demonstrated a sharp increase at Time 2, *F* (1,97) = 35.74, *p* < .001, and a slight decrease at Time 3, *F* (1,97) = 4.91, *p* = .03.

### NSSI severity

Main effects and an interaction between disclosure and time were evident on the severity of NSSI. On average, young people who disclosed their NSSI reported more severe NSSI, *F*(1310) = 6.19, *p* = .01. Severity of NSSI fluctuated over time, decreasing from baseline to Time 2, *F*(1310) = 8.02, *p* < .001, and then increasing at Time 3, *F*(1310) = 2.92, *p* = .01. Among those who disclosed their NSSI, severity of the behaviour decreased from baseline to Time 2, *F*(1143) = 4.41, *p* < .001, then stabilised, *F*(1143) = 1.25, *p* = .64. Among those who did not disclose their NSSI severity decreased from baseline to Time 2, *F*(1,59) = 3.38, *p* = .004, and increased at Time 3, *F*(1,59) = 5.07, *p* < .001.

Among those who disclosed their behaviour, reported NSSI severity changed over time, and an interaction with disclosure to a peer or adult was observed. On average, NSSI severity decreased from baseline to Time 2, *F*(1198) = 3.75, *p* = .001, and remained stable, *F*(1198) = .01, *p* = 1.0. However, this change was only evident among youth who disclosed to adults, *F*(2,50) = 5.76, *p* = .006, with no change over time seen for youth who disclosed to peers, *F*(2146) = 1.48, *p* = .23.

## Discussion

Overall, we found that disclosure of NSSI facilitated further help-seeking from peers, improved coping and reduced suicidality. However, disclosure to peers may reduce perceived social support and encourage NSSI in others. Conversely, confiding in an adult may be an important protective factor, increasing both adaptive coping skills and the belief in the ability to employ alternative coping skills to avoid death by suicide, and possibly reducing the severity of NSSI.

Among young self-injurers, most had disclosed their NSSI to another person, primarily to peers (friends). Preference to disclose to peers is consistent with findings from other studies [13]. When adults were the chosen confidantes, parents were more common than any other adult. We also found young people who disclosed NSSI (to either adults or peers) were more likely to have friends who are self-injurers. Arguably, knowing others who self-injure reduces stigma and facilitates disclosure of NSSI. It is also possible that young people who disclosed to adults had better pre-existing relationships with the adults in their lives, including better home environment. Anticipation of support and non-judgemental attitudes would facilitate disclosure to adults [33]. A more supportive family environment, open communication between parents and their children and modelling of adaptive coping might also explain why youth who disclosed to adults reported better psychosocial functioning over time.

That over 40 % of young people had not disclosed their NSSI to anyone highlights the secretive nature of NSSI and the need to encourage disclosure of the behaviour. Little work has explored the relationship between exposure to NSSI and stigma or attitudes towards people who self-injure. Among school staff, having experience with students who self-injured is associated with more positive attitudes towards those students [9], but among psychologists no relationship exists between years of experience working with self-injuring clients and attitudes towards NSSI [34]. Further work is required to explore the relationship between exposure to NSSI and stigma, with a view to reducing stigma and facilitating help-seeking.

It is concerning that disclosure to others was associated with acquiring new friends who self-injured. There is ongoing concern that discussion of NSSI among peer groups risks an increase in the incidence of NSSI. While there is evidence of contagion in psychiatric settings e.g. [35], less is known about the impact of discussion of NSSI among community-based samples. Previous work suggests that when an adolescent experiences an accumulation of negative life events (e.g. bullying), knowing a friend who self-injures is related to later NSSI [15], a ‘learned coping strategy’. Alternative explanations include the notion that an individual may change friendship groups after beginning to self-injure. This might arise as a result of stigma or disapproval expressed by friends (see discussion below), and a seeking out of like-minded friends who also self-injure. Disclosure to friends might also prompt those friends to disclose their own NSSI, thus increasing the awareness of the number of friends who self-injure. However, the increase in new friends who self-injure was not limited to disclosure to peers, but also observed when youth disclosed to adults. A plausible explanation for this relationship might be that disclosing to an adult could result in being placed into a therapy group with others who self-injure, increasing the number of self-injuring friends. Unfortunately, with the data collected we are not able to ascertain the reason for this effect.

Young people who disclosed to peers as opposed to adults were more likely to seek help by the end of the study period. While this is encouraging, peers remained the primary source of support. Although seeking help from informal sources facilitates formal help-seeking [14], more effort is required to determine how best to support young people to seek help from adults and formal supports (e.g. school psychologist), and reduce help-negation. Turning to peers may stem from fear of stigma, a perception that parents and other adults lack understanding, and may not be able to help [33]. In hindsight, disclosure of NSSI can be viewed favourably by young people who receive help [17]. Capitalising on this through peer support programs may warrant consideration.

There was a general deterioration in level of perceived social support over time among those who disclosed their NSSI, particularly for those who disclosed their NSSI to friends. Although friends are generally supportive, learning of NSSI may make them react negatively (i.e. due to stigma, lack of understanding) and distance themselves. This negative reaction is then reflected over time in a reduction in perceived social support. Even young people who self-harm (broadly defined) express difficulty understanding self-harm in others [17]. However, despite this, help-seeking continued to increase suggesting that perhaps whilst the reactions of peers may not always be positive, overall it did not discourage further help-seeking.

### Limitations and suggestions for future research

First, we explored differences in whether the confidante was a peer or adult, but people who told peers may also have confided in adults; confidantes were not mutually exclusive. Consequently we cannot draw definitive conclusions about the effect of disclosure specifically to adults or peers on later psycho-social functioning. Arguably, the first person that comes to mind when asked about disclosure is likely to be most significant in the disclosure/recovery process, but further work is required to tease apart the differential effects of confiding in peers and adults. Second, although we collected data at three time points over a two year period, our design cannot detect nuances in family or social dynamics that may impact on, or result from, disclosure of NSSI. More detailed examination of the relationships explored here is required. Third, we did not explore the impact of disclosing NSSI on the confidantes, or the way in which confidantes reacted to the disclosure that someone they know self-injured. The reaction of family and friends will impact the young person and their future help-seeking behaviour. Fourth, the measures of disclosure and help-seeking may be confounded, as disclosure of NSSI may be perceived as a form of help-seeking. However, only three participants explicitly stated seeking help for NSSI when asked about the problem they were seeking help for. Fifth, participants included in this study are not representative of the general population of Australian adolescents, limiting generalizability of the findings. Finally, the internal consistency of the ‘reference to others’ subscale of our coping measure was poor, potentially explaining the null results observed with this scale.

## Conclusion

Overall, our results indicate that disclosure of NSSI is a positive behaviour that should be encouraged in young people. Our findings provide potential avenues for combatting help-negation related to NSSI, suggesting information and support initiatives should be directed primarily to the young person’s peer group and to parents as the two priority groups. Parents and friends of young people who self-injure should also be encouraged to model the use of problem-focused coping strategies. Importantly, further work is required to ascertain the reciprocal effects on parents and the family dynamic when a family member self-injures.
